# The Systems of Naringenin with Solubilizers Expand Its Capability to Prevent Neurodegenerative Diseases

**DOI:** 10.3390/ijms23020755

**Published:** 2022-01-11

**Authors:** Anna Stasiłowicz-Krzemień, Michał Gołębiewski, Anita Płazińska, Wojciech Płaziński, Andrzej Miklaszewski, Marcin Żarowski, Zofia Adamska-Jernaś, Judyta Cielecka-Piontek

**Affiliations:** 1 Department of Pharmacognosy, Faculty of Pharmacy, Poznan University of Medical Sciences, Swiecickiego 4, 60-781 Poznan, Poland; astasilowicz@ump.edu.pl (A.S.-K.); golombek112@wp.pl (M.G.); 2 Department of Biopharmacy, Faculty of Pharmacy, Medical University of Lublin, Chodzki 4a, 20-093 Lublin, Poland; anita.plazinska@umLub.pl; 3 Jerzy Haber Institute of Catalysis and Surface Chemistry, Polish Academy of Sciences, Niezapominajek 8, 30-239 Krakow, Poland; wojtek_plazinski@o2.pl; 4 Institute of Materials Science and Engineering, Poznan University of Technology, Jana Pawla II 24, 61-138 Poznan, Poland; andrzej.miklaszewski@put.poznan.pl; 5 Department of Developmental Neurology, Poznan University of Medical Sciences, Przybyszewski 49 Str., 60-355 Poznan, Poland; zarowski@ump.edu.pl; 6 Department of General and Transplantation Surgery, Poznan University of Medical Sciences, Przybyszewski 49 Str., 60-355 Poznan, Poland; adamska.jernas.z@gmail.com

**Keywords:** naringenin, cyclodextrins, neuroprotection, acetylcholinesterase, butyrylcholinesterase, tyrosinase, solubility, permeability, antioxidant, blood-brain barrier

## Abstract

Background: Naringenin (NAR) is a flavonoid with excellent antioxidant and neuroprotective potential that is limited by its low solubility. Thus, solid dispersions with β-cyclodextrin (β-CD), hydroxypropyl-β-cyclodextrin (HP-β-CD), hydroxypropylmethylcellulose (HPMC), and microenvironmental pH modifiers were prepared. Methods: The systems formation analysis was performed by X-Ray Powder Diffraction (XRPD) and Fourier-transform infrared spectroscopy (FT-IR). Water solubility and dissolution rates were studied with a pH of 1.2 and 6.8. In vitro permeability through the gastrointestinal tract (GIT) and the blood-brain barrier (BBB) was assessed with the parallel artificial membrane permeability assay (PAMPA) assay. The antioxidant activity was studied with the 2,2′-azinobis-(3-ethylbenzothiazoline-6-sulfonic acid (ABTS) and cupric ion reducing antioxidant capacity (CUPRAC) assays, while in vitro enzymes studies involved the inhibition of acetylcholinesterase, butyrylcholinesterase, and tyrosinase. For the most promising system, in silico studies were conducted. Results: NAR solubility was increased 458-fold by the solid dispersion NAR:HP-β-CD:NaHCO_3_ in a mass ratio of 1:3:1. The dissolution rate was elevated from 8.216% to 88.712% in a pH of 1.2 and from 11.644% to 88.843% in a pH of 6.8 (within 3 h). NAR GIT permeability, described as the apparent permeability coefficient, was increased from 2.789 × 10^−6^ cm s^−1^ to 2.909 × 10^−5^ cm s^−1^ in an acidic pH and from 1.197 × 10^−6^ cm s^−1^ to 2.145 × 10^−5^ cm s^−1^ in a basic pH. NAR BBB permeability was established as 4.275 × 10^−6^ cm s^−1^. The antioxidant activity and enzyme inhibition were also increased. Computational studies confirmed NAR:HP-β-CD inclusion complex formation. Conclusions: A significant improvement in NAR solubility was associated with an increase in its biological activity.

## 1. Introduction

The aging of society is observed in highly developed countries worldwide. Concurrently, the number of cases of neurodegenerative diseases is rising. Neurodegeneration is a slowly progressing and continuing dysfunction and a loss of axons and neurons within the central nervous system (CNS) [1]. It is the basis of diseases, such as Parkinson’s disease, Alzheimer’s disease, neurotrophic viral infections, multiple sclerosis, traumatic brain injury, and paraneoplastic disorders. Projections show an increase in dementia cases worldwide. According to the World Health Organization (WHO), about 55 million people currently have dementia, and there are nearly 10 million new cases each year [2].

One of the potential substances that might be used as a neuroprotectant in the prevention of neurodegenerative diseases is naringenin (NAR). NAR is a natural flavanone present in citrus fruits, tomatoes, and grapefruits that exhibits a wide range of biological activity. Naringenin exerts neuroprotective, immunomodulatory, anti-tumor, anti-depressant, antiviral, antibacterial, antioxidant, anti-inflammatory, and cardioprotective effects [3,4,5].

NAR is poorly soluble in water. Studies show that the solubility of NAR is in the range of 4.38–46.0 μg/mL, depending on its purity, the conditions of studies, and the methods used [6,7,8]. Ascending temperature gradually increases NAR solubility to 71.85 µg/mL at 37 °C [9]. Water solubility is an essential aspect limiting the bioavailability of the substance, due to the fact that only dissolved compounds can be absorbed from the gastrointestinal tract [10]. The biopharmaceutics classification system (BCS) divides substances into four classes according to their solubility and permeability [11]. Poorly soluble substances can be classified in the II or IV BCS classes. The II class consists of highly permeable active pharmaceutical ingredients (APIs), and poorly permeable APIs are assigned to the IV class. NAR has been classified into the II class of BCS.

Despite an enormous potential of biological activity and possession of high membrane permeability, NAR is poorly bioavailable. The relative cumulative urinary excretion, expressed as a percentage of the administered dose, which can be considered as the oral bioavailability estimator, is only 5.81% [12]. Moreover, NAR is strongly metabolized in the liver and converted into glucuronide intermediates, which decreases its bioavailability [13]. The literature comprises studies attempting to increase the solubility and, therefore, bioavailability by preparing inclusion complexes, self-nanoemulsifying drug delivery systems, solid dispersions, and nanoparticles [8,14,15,16]. A particularly valuable method for improving the solubility of NAR seems to be its combination with cyclodextrins (CDs, oligomers of d-glucopyranose linked by α-1,4 glycosidic bonds) [17,18]. The first pioneering work showed the significant potential for combining NAR with CD when Semalty et al. prepared complexes with β-CD in three different molar ratios (1:1, 1:2, and 1:3) by the solvent evaporation method [19]. As a result, the water solubility increased two times and the maximum release was elevated from 48.78% to 98.0–100%. In another study, the complexation of NAR with hydroxypropyl-β-CD (HP-β-CD) increased NAR solubility by over 400-fold [14]. It also had an 11-fold impact on the permeability of NAR in the Caco-2 model of the gut epithelium [14]. The results were also noticed in studies on rats [14]. The complex improved NAR pharmacokinetic properties. The area under the curve (AUC) values were elevated 7.4-fold, and there was a 14.6-fold increase in the maximum plasma concentration.

Apart from CDs, synthetic polymers, such as Poloxamer 188 or Pluronic F127, are also used to enhance the physicochemical properties of NAR. Amorphous ternary solid dispersions of NAR, Poloxamer 188, and Neusilin US2, prepared by hot-melt extrusion, enabled an increase in the release of NAR within 2 h from 22% to 77% [20]. The NAR-loaded mixed micelle formulation with NAR:Pluronic F127:Tween 80 in a weight ratio of 1:10:0.2, prepared by a thin-film hydration method, increased the NAR solubility 27-fold and also improved the bioavailability in rats from 4.1 to 26.9% [21].

This study aimed to enhance the physicochemical properties and biological activity of NAR by preparation systems with β-CD, HP-β-CD, and hydroxypropylmethylcellulose (HPMC). The studies in the literature that have improved the physicochemical properties of NAR mostly made use of only one excipient in the preparation of the delivery systems. The novelty in our research is the addition of a third component to the binary systems, forming ternary systems. The literature describes pH modulators, surfactants, polymers, and adsorbents as the second excipients to amplify solubility, dissolution rate, and stability or to inhibit recrystallization in the amorphous state [22]. Microenvironmental pH modifiers, magnesium stearate, and sodium bicarbonate (NaHCO_3_) implement a complementary functionality to the binary solid dispersion and have not been used in NAR studies before.

## 2. Results

### 2.1. The Preparation of the Systems

The solid dispersions of NAR with β-CD, HP-β-CD, and HPMC were prepared by the co-precipitation method. Previous studies of NAR presented the preparation of principally binary delivery systems with various excipients, including polymers, by many methods, e.g., solvent evaporation, lyophilization, hot-melt extrusion, spray drying, vacuum drying, and microwave-vacuum drying [19,20,23,24,25]. The literature data mention only one study describing a ternary solid dispersion of NAR with Poloxamer 188 and Neusilin US2. This study’s novelty comprises the use of a microenvironmental pH modifiers, magnesium stearate, and sodium bicarbonate as second excipients that are complementary to the solubilizers.

### 2.2. X-ray Powder Diffraction 

**Figure 1:**
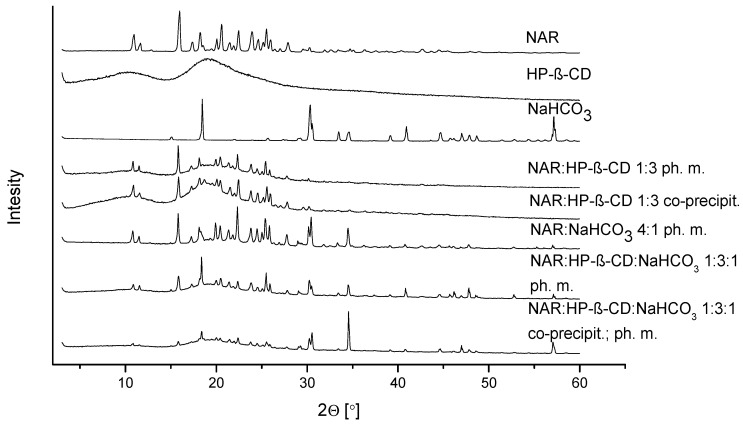
The XRPD diffraction patterns of NAR, HP-β-CD, NaHCO_3_, the binary system (NAR:HP-β-CD) obtained by the co-precipitation method, the physical mixtures (NAR:HP-β-CD; NAR:NaHCO_3_), the ternary system (NAR:HP-β-CD:NaHCO_3_), and the physical mixture (NAR:HP-β-CD:NaHCO_3_).

An X-ray Powder Diffraction (XRPD) study was performed for the greatest solubilizing system and its components. NAR has a crystalline structure. Its diffractogram displays visible sharp reflexes in the range of 2θ from 10° to 30° (Figure 1). In contrast, HP-β-CD is present in the amorphous state, which is shown as an amorphous halo on the diffractogram. The NaHCO_3_ diffractogram has sharp reflexes, exhibiting its crystal nature. The crystallinity of NAR is visible in both physical mixtures of NAR with HP-β-CD and with NaHCO_3_. In the case of systems prepared by the co-precipitation method with HP-β-CD and with both HP-β-CD and NaHCO_3_, the peaks are still present on the diffractogram, proving the crystallinity of NAR.

### 2.3. Fourier-Transform Infrared Spectroscopy

**Figure 2:**
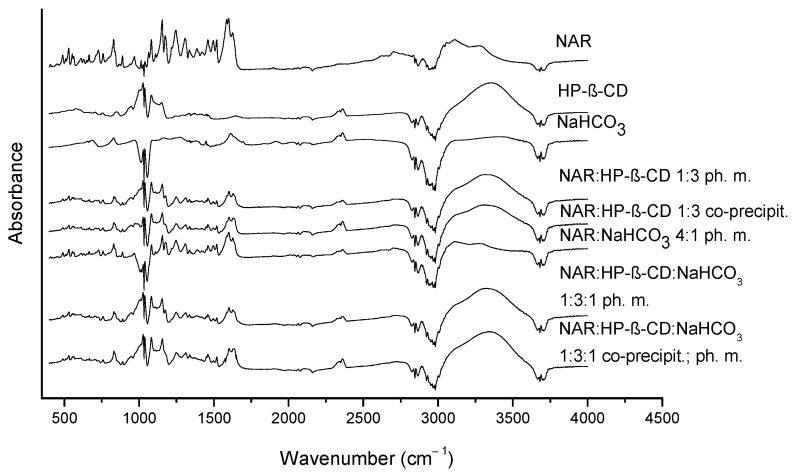
The FT-IR spectra of NAR, HP-β-CD, NaHCO_3_, the binary system (NAR:HP-β-CD) obtained by the co-precipitation method, the physical mixtures (NAR:HP-β-CD; NAR:NaHCO_3_), the ternary system (NAR:HP-β-CD:NaHCO_3_), and the physical mixture (NAR:HP-β-CD:NaHCO_3_).

The NAR identity was confirmed by comparing the FT-IR spectrum of the sample (Figure 2) with the theoretical one (Figure S1). The individual NAR bands are described in a table in the Supplementary Materials (Table S1). A Fourier-transform infrared spectroscopy-attenuated total reflectance (FTIR-ATR) study was performed for the greatest solubilizing system and its components. The HP-β-CD spectrum is characterized by intense bands within the range 1000–1200 cm^−1^ and a broad band at 3354 cm^−1^, associated with the symmetric and antisymmetric O-H stretching vibrations of the HP-β-CD molecule. Within the range of 1200 cm^−1^ to 1500 cm^−1^ bands are present that are associated with the wagging vibration of the CH_2_ and O-H bonds. In the wavenumber of 2500–3000 cm^−1^, there are bands related to the symmetric and antisymmetric stretching vibration of the C-H bond in the HP-β-CD molecule. The bands at 830 cm^−1^ and 687 cm^−1^ in the NaHCO_3_ IR spectrum are associated with the stretching vibration of CO_3_ ^2−^ [26].

The spectra of the binary system of NAR with HP-β-CD and ternary system of NAR with HP-β-CD and NaHCO_3_ that were obtained by the co-precipitation method and the analogous physical mixtures were analyzed. The IR spectra of the binary and ternary systems seem very similar. However, the ternary solid dispersion spectrum comprises additional bands from NaHCO_3_. The spectra of the binary and ternary systems contain a shift of the HP-β-CD band from 3354 cm^−1^ to 3346 cm^−1^ for the ternary system and to 3320 cm^−1^ for the binary system [27]. Such changes were not observed for physical mixtures. The intensity of the characteristic band of NAR at 1600 cm^−1^, associated with the stretching vibration of the C=C in the aromatic ring, decreases in the binary and ternary system spectra [28]. The IR spectrum of NAR comprises bands at 1154 cm^−1^ and at 1245 cm^−1^ related to the bending vibration of the C-O-H bonds and a band at 3688 cm^−1^ corresponding to the stretching vibration of the O-H bond. In the spectra of the binary and ternary systems, obtained by the co-precipitation method, the band at 1175 cm^−1^ broadens and, thus, appears to disappear. The band at 1175 cm^−1^ in physical mixtures is reduced, but it is still visible. The band at 1175 cm^−1^, according to the visualization performed in the GaussView program, is associated with the phenol group of NAR ring A. The bands at 1154 cm^−1^, 1245 cm^−1^, and 3688 cm^−1^ are connected with the phenolic groups in ring C of NAR. These changes suggest hydrogen bond formation between a phenolic OH group of NAR ring A and a hydroxyl group of HP-β-CD. The analysis of the IR spectra indicates that the NAR aromatic ring was included in the inside of the HP-β-CD cavity, and the inclusion complex was formed.

### 2.4. The Solubility Study

**Figure 3:**
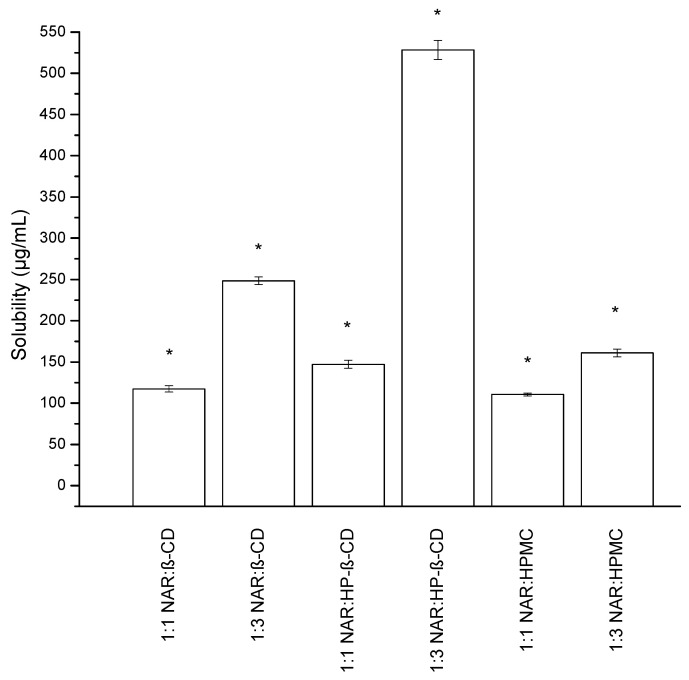
Solubility of NAR in binary solid dispersions prepared by the co-precipitation method. (*) indicates statistically significant differences, *p* < 0.05.

NAR water solubility was determined to be 2.523 ± 0.062 µg/mL. The solubility was increased in the systems with the solubilizers β-CD, HP-β-CD, and HPMC in the ratios of 1:1 and 1:3 (Figure 3). The greatest NAR solubility results were acquired in the systems 1:3 NAR:HP-β-CD, with an almost 210-fold improvement, 1:3 NAR:β-CD, with an almost 99-fold elevation, and 1:3 NAR:HPMC, with an almost 64-fold rise. These three binary systems were enriched with the microenvironmental pH modifiers magnesium stearate and NaHCO_3_.

The ternary systems with local pH modifiers showed even more excellent NAR solubility. The additives to the binary systems were 10% (weight percentage in relation to the total weight of the ternary system) of NaHCO_3_, 20% of NaHCO_3_, and 50% of magnesium stearate. Sodium bicarbonate is used in tablets in the amount of 10–40% as a microenvironmental pH modifier [29]. NaHCO_3_ is commonly used in oral formulations containing various APIs, including, among others, acetylsalicylic acid, bupropion hydrochloride, rosuvastatin calcium, and zolpidem tartrate. Magnesium stearate is a commonly used lubricant used in the pharmaceutical industry. It is present in formulations with acetaminophen, alprazolam, cetirizine hydrochloride, sulfamethoxazole, and others. It is also popular in the food industry as an emulsifier, binder, and thickener and as an anticaking, lubricant, release, and antifoaming agent [30]. In our previous work, magnesium stearate was used as a microenvironmental pH modifier, which enabled the detection of tolfenamic acid during an apparent solubility study and increased the dissolution rate to 63.21% in 180 min in phosphate buffer with a pH of 6.8 [31].

The most significant improvement in NAR solubility was obtained in all ternary systems with a 20% addition of sodium bicarbonate (Table 1). The strongest elevation of NAR solubility was acquired in the system NAR:HP-β-CD:NaHCO_3_, with a solubility of 1156.504 ± 16.163 μg/mL (a 458.3-fold increased solubility). Thus, this system was qualified for further studies.

### 2.5. The Apparent Solubility Study

**Figure 4:**
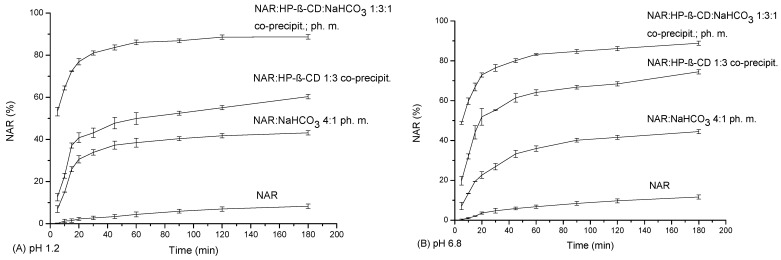
Apparent solubility of NAR, the binary system (NAR:HP-β-CD) obtained by the co-precipitation method, the ternary system (NAR:HP-β-CD:NaHCO_3_), and the physical mixture (NAR:HP-β-CD:NaHCO_3_) in a pH of 1.2 (**A**) and in a pH of 6.8 (**B**).

NAR at an acidic pH showed the maximum dissolution of 8.216 ± 1.063% in the 180 min of the study (Figure 4A). NAR in the binary system with HP-β-CD 1:3 obtained by the co-precipitation method dissolved in the fifth minute to a greater extent than pure NAR after 180 min (12.521 ± 1.756%). The highest result was achieved for this system in 180 min, 60.343 ± 1.063%, which is an almost 5-fold improvement. The NAR physical mixture with NaHCO_3_ 4:1 achieved a maximum result of 43.109 ± 1.286% (3.5 times better than pure NAR). NAR was characterized by the most substantial dissolution profile in the ternary system with HP-β-CD and NaHCO_3_ (1:3:1). In the fifth minute, the dissolution rate was 53.190 ± 2.031%, while it reached a maximum of 88.712 ± 1.013% at 180 min.

In a pH of 6.8, the dissolution profile of NAR was greater than in acidic conditions, according to the weakly acidic nature of NAR (Figure 4B). The pure NAR dissolution profile reached a maximum of 11.644 ± 1.054% at 180 min. NAR in the binary system with HP-β-CD 1:3 showed 19.750 ± 2.131% dissolution at 5 min (pure NAR was only 0.26 ± 0.013%) and reached a maximum of 74.484 ± 1.101% at 180 min. The greatest results were obtained for NAR in the ternary NAR:HP-β-CD:NaHCO_3_ 1:3:1 system. At 5 min, the dissolution reached 48.713 ± 0.667% and at 180 min it reached 88.843 ± 1.315% (more than a 7.5-foldrefinement). In the NAR:NaHCO_3_ 4:1 system, NAR reached a maximum dissolution rate of 44.458 ± 1.211% at 180 min.

### 2.6. Membrane Permeability

**Figure 5:**
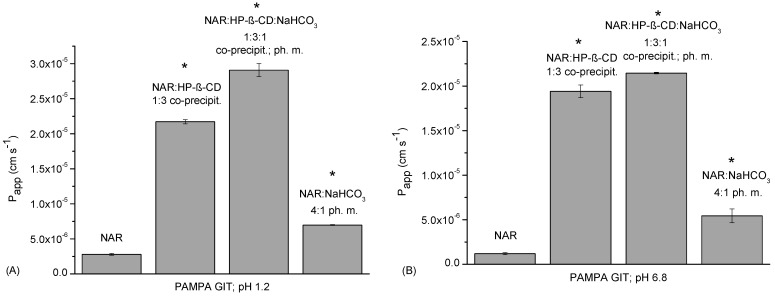
Apparent permeability coefficient values of NAR, the binary system (NAR:HP-β-CD) obtained by the co-precipitation method, the ternary system (NAR:HP-β-CD:NaHCO_3_), and the physical mixture (NAR:HP-β-CD:NaHCO_3_), determined for gastrointestinal permeability in the PAMPA GIT in an acidic pH (**A**) and a basic pH (**B**). (*) indicates statistically significant differences, *p* < 0.05.

NAR permeability was investigated based on the passive diffusion in the parallel artificial membrane permeability assay (PAMPA) system in the gastrointestinal tract (GIT) (Figure 5) and the blood-brain barrier (BBB) model. According to the guidelines, NAR penetrates the GIT membranes well, reaching apparent permeability coefficient (P_app_) values greater than 1.0 × 10^−6^ cm s^−1,^ which is consistent with the literature data [32,33]. At an acidic pH, NAR P_app_ is 2.789 × 10^−6^ ± 1.208 × 10^−7^ cm s^−1^, and in a basic pH it is 1.197 × 10^−6^ ± 1.178 × 10^−7^ cm s^−1^. An approx. 10-fold increase in the permeability of NAR was achieved in both conditions by introducing NAR into the system with HP-β-CD and with HP-β-CD:NaHCO_3_. The most pronounced and statistically significant enhancement of permeability was noticed for the NAR:HP-β-CD:NaHCO_3_ 1:3:1 ternary system, reaching a P_app_ of 2.909 × 10^−5^ ± 9.276 × 10^−7^ cm s^−1^ and 2.145 × 10^−5^ ± 9.106 × 10^−8^ cm s^−1^ in an acidic and basic pH, respectively.

According to the literature data, NAR penetrates well into the CNS, which was confirmed in the PAMPA BBB study, where NAR reached a P_app_ of 4.275 × 10^−6^ ± 1.315 × 10^−6^ cm s^−1^ [34,35]. The guidelines classify compounds with a P_app_ value greater than 4.0 × 10^−6^ cm s^−1^ as highly permeable [36].

### 2.7. Antioxidant Activity

NAR at the maximal achievable concentration after dissolving pure NAR in water (2.52 µg/mL) does not inhibit 2,2′-azino-bis[3-ethylbenzthiazoline-6-sulphonic acid] radical cation (ABTS^•+^) (Table 2). Associating NAR with HP-βCD 1:3, either as a physical mixture or as a system prepared with the co-precipitation method, results in radical inhibitions of 37.43 ± 1.16% and 38.43 ± 1.23%, respectively. The most significant elevation in NAR solubility was obtained for the ternary system with HP-β-CD and NaHCO_3_, which resulted in the most potent antioxidant activity against ABTS^•+^. The 49.45 ± 2.41% inhibition of radicals was achieved for NAR in the ternary NAR:HP-β-CD:NaHCO_3_ 1:3:1 system obtained with the co-precipitation method.

The antioxidant activity of NAR was also tested in cupric ion reducing antioxidant capacity (CUPRAC) assay as the ability to reduce copper ions. NAR at the maximal attainable water concentration of 2.520 μg/mL did not provide a reduction of copper ions (Table 2). As the concentration of NAR increased, its antioxidant activity also elevated. The antioxidant activity for NAR at ph. M. and in the system with HP-β-CD (1:3) caused an increase in activity defined as EC 0.44 ± 0.03 and 0.46 ± 0.04, respectively. The most considerable improvement was achieved for NAR in the ternary system with HP-β-CD and NaHCO_3_, with a value of EC 0.77 ± 0.05.

### 2.8. Inhibition of Enzymes Influencing the Development of Neurodegenerative Diseases

The dependence of the increase in NAR antioxidant activity on the elevation in NAR concentration was confirmed in the literature data by Kang et al. in ABTS assays and ferric reducing antioxidant power (FRAP) assays and by Martinez et al. for scavenging the radical ABTS, ^•^OH, iron-independent lipid peroxidation, and iron-induced lipid peroxidation [37,38,39]. Due to its antioxidant properties, NAR has an excellent potential to act against neurodegenerative mechanisms, as oxidative stress causes the aggregation and loss of protein function, lipid peroxidation, and damage to nucleic acids, cell structures, and their function and excessive chronic inflammation. The amount of damage accumulates with age and can lead to progressive neurodegeneration. The cerebrum is especially sensitive to damage, due to the richness of lipids in its structure. The relationship between oxidative stress and the development of Parkinson’s and Alzheimer’s diseases has been proven [40,41]. The inhibitory activity against enzymes influencing the development of these diseases is also essential. Thus, the potential of NAR inhibitory activity against acetylcholinesterase (AChE), butyrylcholinesterase (BChE), and tyrosinase was investigated.

**Figure 6:**
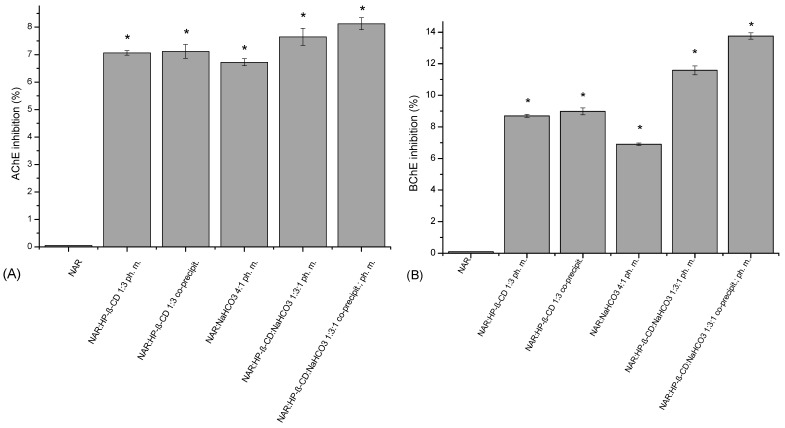
AChE (**A**) and BChE (**B**) inhibitory activity of NAR, the binary system (NAR:HP-β-CD) obtained by the co-precipitation method, the physical mixtures (NAR:HP-β-CD; NAR:NaHCO_3_), the ternary system (NAR:HP-β-CD:NaHCO_3_), and the physical mixture (NAR:HP-β-CD:NaHCO_3_). (*) indicates statistically significant differences, *p* < 0.05.

NAR did not provide inhibition of AChE at the concentration of 2.520 μg/mL (Figure 6). Preparation of the physical mixture of a system with HP-β-CD in the ratio of 1:3 increased the water concentration of NAR (to 506.29 μg/mL and 542.11 μg/mL) and increased AChE inhibition to 7.06 ± 0.08% and 7.12 ± 0.25%. The second excipient NaHCO_3_ added to the binary system elevated NAR solubility (to 1156.50 μg/mL) and raised enzyme inhibition up to 8.12 ± 0.22%.

NAR at the concentration of 2.520 μg/mL did not inhibit BChE (Figure 6). However, ph. m. or a system with HP-β-CD in the ratio 1:3, by elevating the water concentration of NAR to 506.29 μg/mL and 542.11 μg/mL, allowed enzyme inhibitions of 8.70 ± 0.15% and 8.98 ± 0.22%, respectively, to be reached. The addition of NaHCO_3_ to the binary system (NAR:HP-β-CD:NaHCO_3_ 1:3:1) caused the increase in NAR solubility to 1156.50 μg/mL resulting in the most significant BChE inhibition, at the level of 13.75 ± 0.28%.

**Figure 7:**
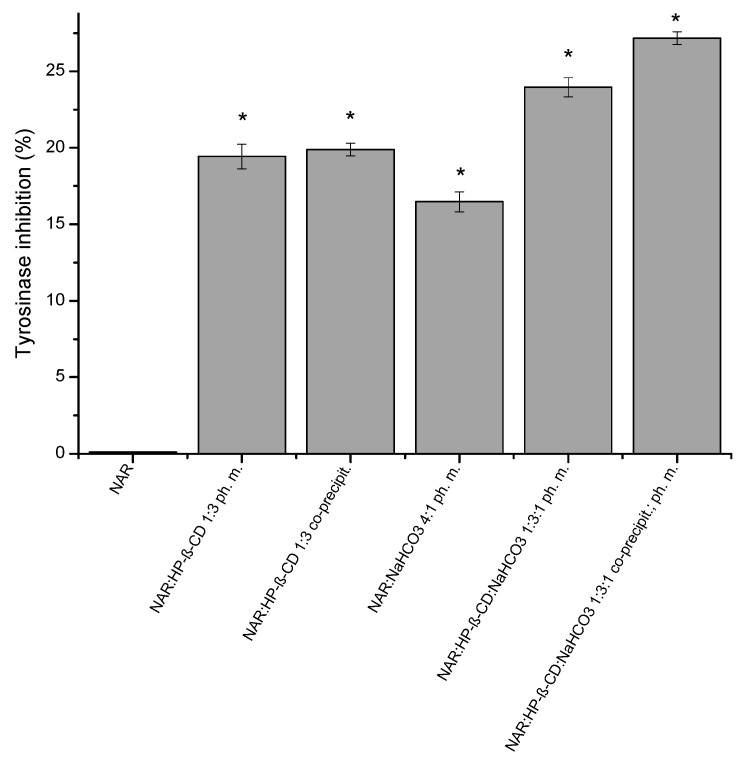
Tyrosinase inhibitory activity of NAR, the binary system (NAR:HP-β-CD) obtained by the co-precipitation method, the physical mixtures (NAR:HP-β-CD; NAR: NaHCO_3_), the ternary system (NAR:HP-β-CD:NaHCO_3_), and the physical mixture (NAR:HP-β-CD:NaHCO_3_). (*) indicates statistically significant differences, *p* < 0.05.

Tyrosinase inhibition results were also concentration-dependent. NAR at the concentration of 2.520 μg/mL did not affect the enzyme (Figure 7). The addition of HP-β-CD in the ratio of 1:3 for the ph. m. or the system caused the increase of inhibition to 19.44 ± 0.35% and 19.89 ± 0.69%, respectively. Ternary ph. m. or system containing NAR:HP-β-CD:NaHCO_3_ 1:3:1 raised the tyrosinase inhibition to 23.96 ± 0.38% and 27.17 ± 0.79%, respectively.

### 2.9. Docking

The molecular modeling investigations were restricted to NAR-HP-β-CD complexes, as they showed the most promising results, as described in the previous subsections. As described in detail in the Materials and Methods, the unknown quantitative patterns of chemical substitution required the consideration of the two distinct structures of HP-β-CD molecules. The main aim of this aspect of the study was to characterize the structure of the NAR- HP-β-CD complex and the molecular interactions underlying its formation.

Both types of complexes that were studied exhibit fairly similar orientations of the NAR molecule in the binding cavity of HP-β-CD. Namely, we have obtained an expected guest-host complex, with the NAR molecule located in the center of the binding cavity formed by the inner channel of HP-β-CD. All observations given below rely on the inspection of multiple binding poses recovered during the docking study and characterized by only minor differences in binding energies (below 0.5 kcal/mol). The dihydroxyphenyl moiety of NAR is directed towards that edge of the HP-β-CD torus, which contains only the hydroxyl (or hydroxyl and HP) groups. On the opposite, phenolic moiety is located closer to the hydroxymethyl groups and ring oxygen atoms of β-CD. Among the chemical groups composing the guest molecule, the most intensive interactions seem to be created by the phenolic moiety interacting with hydrophobic patches on the inner surface of the binding cavity of HP-β-CD. At the same time, the dihydroxyphenyl group of NAR interacts with either hydroxyl or 2HP groups of HP-β-CD, depending on the substitution type. In spite of the maintained general pattern of the guest-host interactions (as described above), there exist a number of minor differences between the observed contacts, which mainly involve the most flexible groups of HP-β-CD, i.e., the HP, hydroxymethyl, and hydroxyl moieties. The interactions with such groups (involving intermolecular hydrogen bonding) are not maintained across the whole set of low-energy structures. A relatively low scatter in the determined binding energies, accompanied by notable structural differences, suggests the high flexibility of the whole molecular complexes, independent of the considered substitution pattern.

The calculated binding energies vary between −6.5 and −6.0 kcal/mol (substitution pattern 1) and between −7.1 and −6.6 kcal/mol (substitution pattern 2). Both these ranges indicate strongly favorable interactions between HP-β-CD and NAR.

### 2.10. Molecular Dynamics

The structural topology of the NAR-HP-β-CD complexes obtained during docking, as well as most of the types of identified intermolecular contacts, were maintained during molecular dynamics (MD) simulations. More precisely, dihydroxyphenyl moiety is still directed towards the edge of the 2HP-β-CD molecule, which contains only the hydroxyl (or hydroxyl and 2HP) groups. The opposite, phenolic moiety is located in the proximity of the hydroxymethyl groups and ring oxygen atoms of β-CD. In the case of both substitution patterns, the most characteristic and conserved contact type is delivered by the aromatic moieties of the guest molecule interacting with the inner part of the 2HP-β-CD cavity. From the chemical nature of the interacting molecular fragments, one can deduce the existence of the CH-π attractive interactions. Although molecular mechanics force fields do not contain the explicit functional forms responsible for reproducing such interactions, the combination of non-bonded (Lennard-Jones and Coulombic) interactions is fully capable of effectively mimicking them [42].

**Figure 8:**
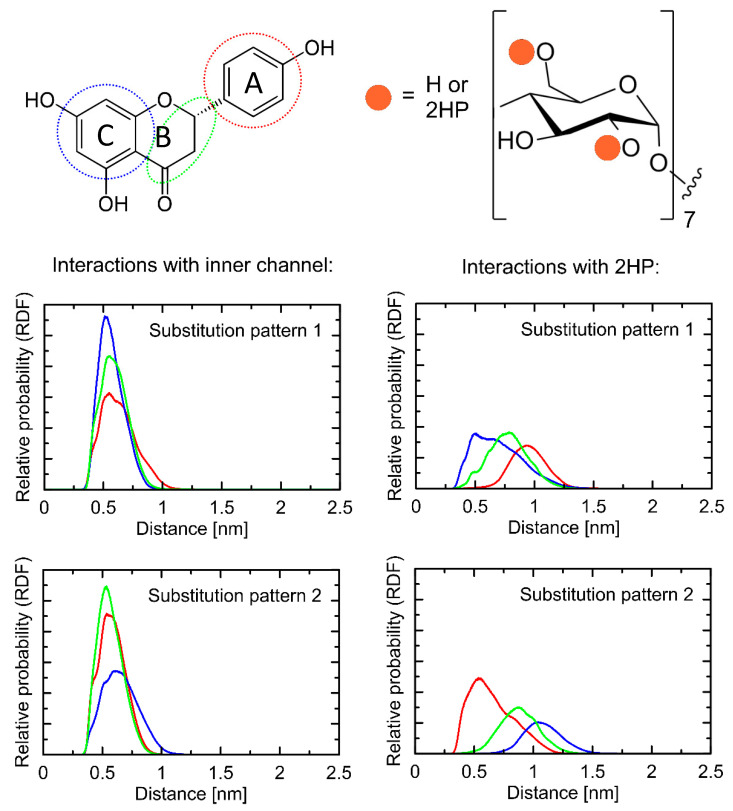
RDFs calculated for the distances between selected fragments of the studied systems. In the case of HP-β-CD, both the inner channel (aliphatic carbon atoms within carbohydrate rings) and 2HP moieties were distinguished, whereas the molecule of NAR was considered as the three separate groups. The color code is maintained throughout the figure.

**Figure 9:**
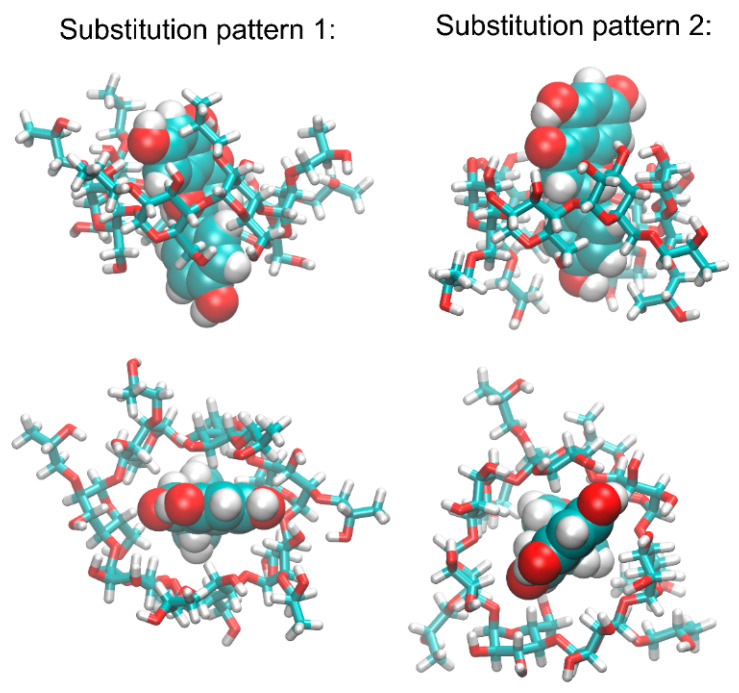
The exemplary structures of the NAR-HP-β-CD complexes, generated during MD simulations (view along two different axes). The two alternative substitution patterns of β-CD were considered.

Figure 8 contains the radial distribution functions (RDFs) illustrating the contacts present in the studied guest-host complexes. The position of the two aromatic rings is altered in comparison to the structures resulting from the docking studies. In the case of both substitution patterns, intensive interactions are expected in the case of all parts of the NAR molecule and the inner channel of HP-β-CD binding cavity. Regarding interactions associated with the two alternative substitution patterns, only minor differences are observed between them. Namely, either contacts with the dihydroxyphenyl group (substitution pattern 1) or phenolic moiety (substitution pattern 2) are favored. Although no specific and attractive interactions can be distinguished in the case of the inner cavity of HP-β-CD and central part of NAR molecule, these two fragments also exhibit intensive contacts, probably due to opportunistic consequences of stronger, CH-π interactions with limiting fragments of the NAR molecule, supported by hydrophobic interactions. Much more diverse RDFs are observed for the 2HP moieties interacting with the NAR molecule. Here, the most intensive contacts are determined by the topology of a given substitution type. Namely, with the position of the guest molecule roughly fixed with respect to the ‘core’ of 2HP-β-CD, the 2HP groups interact with those fragments of the NAR molecule that are located in close proximity. This corresponds either to the dihydroxyphenyl (substitution pattern 1) or phenolic (substitution pattern 2) moieties of NAR. The exemplary snapshots of the MD trajectory are illustrated in Figure 9.

Moreover, the large flexibility of the complex, assumed on the basis of the docking results, is fully confirmed by the significant variation associated with the RDF peaks (Figure 8). The NAR-HP-β-CD complexes are not rigid but display a high degree of conformational heterogeneity, including both the NAR molecule (reorientations around the -C-C- rotatable bond are observed) and the whole complex. A visual inspection of the trajectory reveals that the identified contact types can involve various chemically identical carbohydrate residues across the 2HP-β-CD perimeter.

In spite of configurational feasibility (i.e., high proximity of the 2HP, hydroxymethyl, and hydroxyl groups of HP-β-CD and the hydroxyl and carbonyl groups of NAR), hydrogen bonds between guest and host molecules are very scarce and correspond to 1.028 and 0.556 occurrences per timeframe for substitution patterns (1) and (2), respectively. This can be explained by the influence of the water molecules surrounding the guest-host complexes and saturating the hydrogen-bond donors and acceptors present in the molecules of both HP-β-CD and NAR.

The analysis of some structural features was complemented by the inspection of the energies involved in stabilizing the NAR-HP-β-CD complexes. Although these energies cannot be directly associated with the binding free energies (and, thus, guest-host affinities), they also indicate a strong, favorable association. The magnitude of energies corresponding to short-range, non-bonded interactions between NAR and HP-β-CD are equal to −31.1 kcal/mol (including a 20% contribution of Coulombic interactions) and −28 kcal/mol (including a 15% contribution of Coulombic interactions), for substitution patterns 1 and 2, respectively. A relatively minor contribution of the electrostatic component suggests that the binding process is driven through solvent effects (e.g., minimizing the area of the non-polar surface) or other non-polar interactions (e.g., CH-π interactions, mentioned above).

Finally, the calculations of the average values of the SASA (solvent-accessible surface area) parameter gave extremely similar values for both types of substitution, i.e., 15.8 nm^2^ and 15.5 nm^2^ for patterns 1 and 2, respectively. This speaks for equally favorable spatial accommodation of the guest molecule in the host binding cavity.

Note that the above analysis, considering the two alternative substitution patterns, has no aim to identify the most favorable binding mode or to discriminate between those two possibilities. In the case of real systems, the exact substitution pattern is unknown. Thus, any of these two above possibilities can occur, as well as their combination, or yet another substitution pattern as the intermediate between the limiting cases discussed here. Nevertheless, a large number of similar binding parameters determined for structurally different HP-β-CDs, allows us to assume that the obtained binding characteristics is a fair representation for more diverse systems.

## 3. Discussion

The solid dispersions of NAR with β-CD, HP-β-CD, and HPMC were prepared by the co-precipitation method. The novel approach was the addition of a second excipient, a microenvironmental pH modifier, magnesium stearate, and NaHCO_3,_ which have never been used in NAR ternary solid dispersions before.

Identification studies were performed for the most efficacious system for increasing NAR solubility (NAR:HP-β-CD and NaHCO_3_ in the mass ratio 1:3:1) to reveal the molecular interactions responsible for the increase. NAR is present in the crystalline state in the system with HP-β-CD and NaHCO_3,_ which was proved by the XRPD study. The enhancement in NAR solubility was possible due to the inclusion of a flavonoid molecule into the cyclodextrin cavity. In the empirical FT-IR study, disappearing bands associated with the hydroxyl bonds of NAR and HP-β-CD and a decrease in the intensity of the band connected with the aromatic ring of NAR were observed. This indicates the formation of an inclusion complex of NAR with HP-β-CD. Some of the hydrogen bonds dissociate at a microenvironmental basic pH created by NaHCO_3_, which results in higher solubility of the ternary solid dispersion than for the binary solid dispersion. The increasing NAR solubility after adding NaHCO_3_ to the system indicates a higher probability of the formation of a complex involving HP-β-CD molecules with substitution pattern 2, which is confirmed by FTIR-ATR analysis.

The data in the literature are consistent with the poor NAR solubility—below 100 μg/mL. However, the exact values of the solubility vary from a few to several dozen micrograms per milliliter [6,7,9]. Due to the unfavorable NAR physicochemical properties, attempts to improve them with cyclodextrins are present in the literature. This study presents an increased NAR solubility of 1156.504 ± 16.163 μg/mL, which is a 458.3-fold increased value according to the preparation of a ternary solid dispersion with HP-β-CD and NaHCO_3_ in the mass ratio of 1:3:1. These auxiliary substances are considered safe and are used in many drug formulations available on the market worldwide. They are not absorbed within the GIT and do not reach the bloodstream.

Considering the time required to obtain 80% of the NAR dissolution rate and the maximum value that was obtained in acidic (88.712 ± 1.013%) and in basic conditions (88.843 ± 1.315%), our results are comparable or greater than the results present in the literature. Wang et al. presented solid dispersions of NAR and mannitol prepared by the solvent-evaporation method with various drying methods, including vacuum drying, microwave-vacuum drying, and spray drying [25]. The most significant results in the dissolution test were obtained for the spray-dried solid dispersion, reaching about 80% of NAR in 60 min and a maximum of about 83–84% in 90 min. In another study, the preparation of a solid dispersion of NAR and poloxamer 188 and neusilin US2, prepared by hot-melt extrusion, enabled the enhancement of the dissolution rate of NAR [20]. The greatest results in the apparent solubility study performed in water at 37 ± 0.5 °C were obtained for a solid dispersion with 20% of NAR, 77% of NAR was dissolved in 120 min. A nanodispersion system of polyvinylpyrrolidone/NAR–hesperetin, prepared by the solvent-evaporation method, enabled 100% of NAR to be obtained from a capsule in 100 min in a study performed in phosphate buffer (pH 6.8) at a temperature of 37 ± 0.5 °C [43]. NAR is classified as a highly permeable substance, which was confirmed during permeability studies. NAR permeability through the GIT increased in the NAR:HP-β-CD with NaHCO_3_ system. The P_app_ value increased from 2.789 × 10^−6^ ± 1.208 × 10^−7^ cm s^−1^ to 2.909 × 10^−5^ ± 9.276 × 10^−7^ cm s^−1^ in acidic conditions and from 1.197 × 10^−6^ ± 1.178 × 10^−7^ cm s^−1^ to 2.145 × 10^−5^ ± 9.106 × 10^−8^ cm s^−1^ in basic conditions. The BBB permeability of NAR was assessed, reaching a P_app_ of 4.275 × 10^−6^ ± 1.315 × 10^−6^ cm s^−1^, which is consistent with the literature data [34,35]. The BBB permeability was not assayed for the solid dispersion systems, as they will not leave the gastrointestinal tract. However, it is believed that a higher concentration of NAR in the bloodstream will contribute to a greater amount of NAR crossing BBB. It is well-known that only dissolved substances can be absorbed in the digestive tract. The NAR:HP-β-CD:NaHCO_3_ solid dispersion increased the solubility and dissolution rate of NAR, which promises to achieve a higher concentration of NAR on the other side of the intestinal barrier. This is also supported by an increased permeability through the GIT membranes. These results foster the belief that a later increase of NAR bioavailability could contribute to the protection of nerve cells, which are particularly sensitive to pro-oxidative factors. These considerations are supported by the results of studies on other substances in the literature. Chen et al. used a cyclodextrin metal-organic framework to improve the solubility of isosteviol and to enhance the bioavailability in male Sprague-Dawley rats by 8.67-fold [44]. Wu et al. increased the solubility of biochanin A, according to the micelles preparation with Pluronic F127 and Plasdone S630 [45]. Moreover, the permeability across a Caco-2 cell monolayer from the apical side to the basolateral side was elevated by 54%, and the relative oral bioavailability in Sprague-Dawley rats was increased 2.16-fold. In another study, a flavone cirsiliol was included in β-cyclodextrin, which elevated its solubility 2.1-fold [46]. The cytotoxic effect against cancer cell lines was maintained or elevated, and the tumor growth inhibition studied in mice was increased 1.5-fold. In our previous study, the preparation of the tolfenamic acid system with methyl-β-CD and magnesium stearate caused an increase in the dissolution rate of the API and enhanced the analgesic effect in an animal model of migraine pain [31].

NAR exhibits a strong antioxidant potential. In ABTS and CUPRAC assays, at maximum water concentration (2.52 µg/mL), NAR did not show any antioxidant activity. However, considering the maximum NAR water concentration obtained in the ternary solid dispersion, i.e., 1156.50 µg/mL, NAR inhibited the ABTS radical at 49.45 ± 2.41%, almost reaching the IC_50_ (1202 ± 27.3 µg/mL). In the CUPRAC assay, it reached an EC of 0.77 ± 0.05 (NAR IC_0.5_ 611 ± 22 µg/mL). NAR is a weaker antioxidant than the reference vitamin C, in which the ABTS IC_50_ is 71 ± 3 µg/mL and the CUPRAC IC_0.5_ is 75 ± 4 µg/mL.

The enzymatic studies prove that naringenin may be used in the prevention of neurodegenerative diseases in the future. In Alzheimer’s disease, there is a relative deficiency of acetylcholine, present mainly in neuromuscular plates and synapses of the cholinergic type [47]. Acetylcholinesterase is an enzyme responsible for the breakdown of acetylcholine and some choline esters. NAR dissolved in DMSO has the ability to inhibit AChE with an IC_50_ level of 12.682 ± 0.381 (mg/mL). The reference, galantamine shows an IC_50_ of 0.021 ± 0.001 mg/mL. Butyrylcholinesterase is an enzyme capable of hydrolyzing ester bonds in choline esters. NAR dissolved in DMSO has the ability to inhibit BChE with an IC_50_ level of 6.999 ± 0.142 (mg/mL). Its ability to inhibit BChE is 50 times weaker than galantamine (IC_50_ = 0.140 ± 0.001 mg/mL), which is a powerful inhibitor. Considering the maximum NAR water concentration obtained in ternary solid dispersion (1.156 mg/mL), AChE was inhibited by 8.12 ± 0.22% and BChE was inhibited by 13.75 ± 0.28%. Tyrosinase is an enzyme, containing copper as a cofactor, that is involved in the synthesis of melanin [48]. It catalyzes the metabolism of tyrosine to L-DOPA and L-DOPA to dopaquinone. Blocking the access of the L-DOPA substrate to the active site of this enzyme is a key response to inhibit the degradation of dopamine, and a mismatch in this process occurs in Parkinson’s disease. NAR dissolved in DMSO inhibits tyrosinase, with an IC_50_ of 4.615 ± 0.089 (mg/mL). Its ability to inhibit tyrosinase was stronger than azelaic acid (IC_50_ = 5.883 ± 0.023 mg/mL). NAR in its maximum achievable water concentration in ternary solid dispersion (1.156 mg/mL) inhibited tyrosinase by 27.17 ± 0.79%.

Similar to in vitro assays, in silico studies were performed for the best solubility enhancer, HP-β-CD, to confront the FT-IR analysis. The possible mechanisms of complex formation and its structural features were analyzed according to computational methods, such as docking, and molecular dynamics simulations. In silico results are consistent with FT-IR analysis. Both methods indicate hydrogen bond formation between host and guest of the complex and that NAR inclusion in the HP-β-CD cavity is favored in substitution pattern 2. Thus, molecular interactions underlying the improvement of NAR’s physicochemical parameters and affecting biological activity were explained.

Summarizing, due to our encouraging in vitro results, it is worth investigating the influence of the NAR:HP-β-CD:NaHCO_3_ ternary solid dispersion on the NAR pharmacokinetics, as well as determining of the influence on the prevention or activity in neurodegenerative diseases, such as Alzheimer’s or Parkinson’s diseases, in animal models.

## 4. Materials and Methods

### 4.1. Materials

NAR (purity >95%), 2-hydroxypropyl-β-cyclodextrin (molar substitution 0.8), β-cyclodextrin, and sodium bicarbonate were purchased from Sigma-Aldrich (Poznan, Poland), HPMC was provided by Shin-Etsu Chemical (Tokyo, Japan). Methanol (high-performance liquid chromatography [HPLC] grade) was provided by Merck KGaA (Darmstadt, Germany) and other chemical reagents, including hydrochloric acid, dimethyl sulfoxide, sodium chloride, and potassium dihydrogen phosphate, were obtained from Avantor Performance Materials (Gliwice, Poland). Prisma HT, GIT lipid solution, and acceptor sink buffer were supplied by Pion Inc (Forest Row, East Sussex, England).

### 4.2. Methods

#### 4.2.1. The Preparation of the Systems

NAR systems with β-CD, HP-β-CD, and HPMC in a mass ratio of 1:1 and 1:3 were prepared by the co-precipitation method. NAR (80.0 mg) was dissolved in methanol (10.0 mL), and the polymers (80.0 mg or 240.0 mg) were dissolved in distilled water (the volume depended on the weight and the type of polymer). The solutions were mixed together and stirred at 40 °C at 50 rpm for 24 h until the solvents were evaporated. The resulting systems were gently and shortly (less than 30 s) ground in a mortar to obtain a powder. The binary systems were stored in a desiccator until the dissolution test was performed.

Then, the three systems associated with the greatest increase in NAR solubility (1:3 NAR:HP-β-CD; 1:3 NAR:β-CD; and 1:3 NAR:HPMC) were selected. Ternary systems with local pH modifiers (10% or 20% (*w*/*w*) of NaHCO_3_, or 50% (*w*/*w*) of magnesium stearate) were prepared by grinding the binary systems with the third component in a mortar. The systems were stored in a desiccator.

#### 4.2.2. Fourier-Transform Infrared Spectroscopy

The FTIR-ATR spectra were performed on an IRTracer-100 spectrophotometer (Kyoto, Kyoto Prefecture, Japan). All spectra were recorded within the frequency range of 4000 and 400 cm^−1^ in the absorbance mode. The apparatus was set for the following parameters: resolution, 4 cm^−1^; number of scans, 400; apodization, Happ-Genzel. All of the samples were placed on the ATR crystal and then pressed against the ATR crystal while the ATR-FT-IR spectrum was scanned. The spectra of NAR, the excipients, and the systems were compared and analyzed. Identification, intensity, and location of bands on IR spectrum of NAR was performed based on comparison with the FT-IR theoretical spectrum, which was obtained by using density functional theory (DFT). The geometry was optimized using DFT with Becke, three-parameter, Lee-Yang-Parr hybrid functional, and 6–311G(d,p) basis set. DFT calculations were performed using the PL-Grid platform (website: www.plgrid.pl, accessed on 5 December 2021) and the Gaussian 09 package (Wallingford, CT, USA) [49,50]. The GaussView (Wallingford, CT, USA, Version E01) program was used to propose an initial geometry of the investigated molecules and for visual inspection of the normal modes [51]. The analysis of the obtained spectra was carried out using the Origin Pro 8 software (OriginLab Corporation, Northampton, MA, USA).

#### 4.2.3. X-ray Powder Diffraction

The analysis of the crystallinity state of NAR, HP-β-CD, NaHCO_3_, and the systems was performed via the XRPD method. Diffraction patterns were measured on a PANalitycal Empyrean diffractometer with CuK_α_ radiation (1.54056 Å) at a tube voltage of 45 kV and a tube current of 40 mA. The angular range was 3° to 50° with a step size of 0.017° and a counting rate of 15 s/step. The OriginPro 8 software was used to analyze the acquired data [52].

#### 4.2.4. Chromatographic Conditions

**Figure 10:**
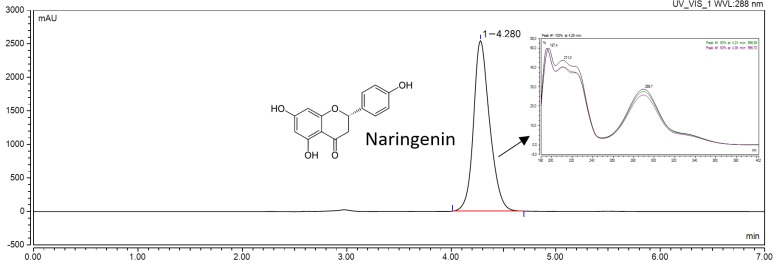
The chromatogram of NAR.

The samples collected from the in vitro, solubility, apparent solubility, and permeability studies were analyzed using ultra-high-performance liquid chromatography with the diode array detector (UHPLC-DAD) method (Dionex Thermo Fisher Scientific, Waltham, MA, USA). The NAR determination was performed using a Phenomenex-C18 column (250 mm × 4.6 mm; 5 µm) as the stationary phase and methanol and water (70:30, *v*/*v*) as the mobile phase [53]. The flow rate of the mobile phase was 1.0 mL/min. The analysis was performed with the column temperature set at 30 °C, the injection volume set at 10.0 µL, and the detection wavelength set at 288 nm. The duration time of the analysis was 7 min, and the retention time of NAR was approximately 4.280 min. The chromatographic data were recorded and processed by Chromeleon software version 7.0 (Thermo Fisher Scientific, Waltham, MA, USA) (Figure 10).

#### 4.2.5. The Solubility Study

The dissolution test was performed at 298.15 K. Excess amounts of NAR and analogical excess amounts of NAR in the systems were added to 4 mL of distilled water and agitated for 24 h at a constant speed of 75 rotations per minute (rpm) using a laboratory incubator MaxQ 4450 (Thermo Scientific, Waltham, MA, USA). Then, the suspensions were filtered through a 0.22 μm filter and analyzed by the HPLC method. All measurements were performed in triplicate.

#### 4.2.6. The Dissolution Rate

An apparent solubility study was performed in the paddle apparatus. NAR (10.0 mg) and the systems (containing 10.0 mg of NAR) were weighed into gelatin capsules and implemented to springs for floating prevention. The test was carried out for 180 min at a pH of 1.2 and 6.8, simulating the gastric and intestinal environments. The vessels were filled with 500 mL of media containing 0.1N HCl (pH 1.2) and the phosphate buffer, which provided a pH of 6.8. The temperature in the vessels was constant, at 310.15 K, and the rotation speed was 50 rpm. Samples with a volume of 5 mL were withdrawn and instantly substituted with an equivalent volume of the temperature-equilibrated fresh medium at the defined time intervals. Then, the samples were filtered through a 0.22 μm membrane filter and analyzed by HPLC. The two-factor values, *f*_1_ and *f*_2_, introduced by Moore and Flanner [54] were used to analyze the dissolution profiles using the following equations:

$$f_{1}=\frac{\sum_{j=1}^{n}\left|R_{j}-T_{j}\right|}{\sum_{j=1}^{n}R_{j}}\;f_{2}=50\times \log\left({\left(1+\left(\frac{1}{n}\right)\overset{n}{\underset{j=1}{\sum }}{\left|R_{j}-T_{j}\right|}^{2}\right)}^{-\frac{1}{2}}\times 100\right)$$

#### 4.2.7. Membrane Permeability

The permeability through biological membranes based on the passive diffusion mechanism was tested using the PAMPA model. The study was carried out in the GIT model (pH of 1.2 and 6.8) and BBB (pH of 7.4). The assay was performed in a sandwich consisting of two 96-well microfilter plates, called donor (at the bottom) and acceptor chambers (at the top), separated by a 120 μm-thick microfilter disc coated with a 20% (*w*/*v*) dodecane solution of a lecithin mixture (Pion Inc., Billerica, MA, USA). The samples of NAR and the systems were prepared equally for the dissolution study. The excess amounts of NAR, the binary system of NAR:HP-β-CD 1:3, the ternary system of NAR:HP-β-CD:NaHCO_3_ 1:3:1, and the physical mixture of NAR:NaHCO_3_ 4:3 were shaken in water for 24 h. The filtrates were used in the permeability assay to investigate how NAR penetrates from aqueous solutions across biological membranes. Bearing in mind that neither CD nor NaHCO_3_ will leave the gastrointestinal tract, the BBB permeability was only studied for NAR. The samples were added to the donor compartments. The chambers were stacked in sandwiches and incubated for 3 h for the GIT model and 4 h for the BBB model in a humidity-saturated atmosphere with the temperature set at 310.15 K. At specific time intervals, the plates were separated to investigate the NAR concentrations using the HPLC-DAD method. The P_app_ was calculated using the equations:

$$P_{\mathrm{app}}=\frac{-ln\left(1-\frac{C_{A}}{C_{equilibrium}}\right)}{S\times \left(\frac{1}{V_{D}}+\frac{1}{V_{A}}\right)\times t}\;C_{equilibrium}=\frac{C_{D}\times V_{D}+C_{A}\times V_{A}}{V_{D}+V_{A}}$$

where *V_D_* is the donor volume, *V_A_* is the acceptor volume, *C_equilibrium_* is the equilibrium concentration $C_{equilibrium}=\frac{C_{D}\times V_{D}+C_{A}\times V_{A}}{V_{D}+V_{A}}$, *S* is the membrane area, and *t* is the incubation time (in seconds). Compounds with a P_app_ in the GIT model below 0.1 × 10^−6^ cm/s are classified as low permeable, substances with 0.1 × 10^−6^ cm/s ≤ P_app_ < 1 × 10^−6^ cm/s are described as medium permeable, and compounds found as highly permeable have a P_app_ ≥ 1 × 10^−6^ cm/s [33]. Compounds whose P_app_ in the BBB model is <2.0 × 10^−6^ cm/s are classified as low permeable. Substances with questionable permeability have P_app_ values in the range of 2.0 to 4.0 × 10^−6^ cm/s. Compounds with high permeability have a P_app_ value at the level > 4.0 × 10^−6^ cm/s [36].

#### 4.2.8. Antioxidant Activity

For all biological activity tests, it was planned to use NAR and prepared systems (1:3 NAR:HP-β-CD (co-precip.), 4:1 NAR:NaHCO_3_ (grinding), and 1:3 NAR:HP-β-CD:NaHCO_3_ (co-precip. and grinding), dissolve them in water and perform assays on their solutions. However, due to the very high antioxidant activity of NaHCO_3_ itself, and in fear of its strong influence on the results of the enzyme inhibition studies, this approach was abandoned. Both cyclodextrin and the local pH modifier NaHCO_3_ were used to influence the solubility of NAR. Since neither cyclodextrin nor NaHCO_3_ will enter the bloodstream, they will not have any effects outside the gastrointestinal tract, and they would have a false impact on the final results, a different method was chosen. Graphs of the dependence of biological activity on the concentration of NAR were prepared, and the exact results were determined for the concentrations resulting from the solubility test of NAR and NAR in the systems. On this basis, the biological activity of pure NAR and NAR activity resulting from the preparation of the systems was determined.

The antioxidant activity of NAR was defined by the CUPRAC assay, according to Apak et al. with modifications [55]. During the study, neocuproin and copper ion (II) complex interacted with antioxidants. During the study, phenolic groups of polyphenols oxidized to quinones, and the neocuproin and copper (II) ion complex (bluish) was reduced to the neocuproin and copper (I) ion complex (yellow). The CUPRAC reagent was prepared from equal volumes of acetate buffer at a pH of 7.0, a 7.5 mM ethanolic 96% solution of neocuproine, and a 10 mM solution of CuCl_2_·H_2_O. The NAR concentrations for the assay were prepared in the range of 1.0 μg/mL to 2.5 mg/mL in DMSO. During the study, 50 µL of the NAR solution and 150 µL of the CUPRAC reagent were applied to a 96-well plate, incubated with shaking at room temperature, and protected from light for 30 min. After incubation, the absorbance (EC) was measured at the wavelength set at 450 nm (Multiskan GO, Thermo Fisher Scientific, Waltham, MA, USA). Vitamin C was used as a standard reference. The IC_0_._5_ value corresponds to the NAR concentration indicating 0.5 absorbance.

Another method of determining antioxidant activity is the ABTS method, performed according to Re et al. with modifications [56]. The green cation radical is generated upon the loss of an electron by the nitrogen atom of ABTS caused by potassium persulfate. After introducing a pre-formed radical cation to the antioxidant, the ABTS radical cation is reduced and converted back to its colorless neutral form. The NAR concentrations for assay were prepared in the range of 1.0 μg/mL to 2.5 mg/mL in DMSO. The assay was performed on the 96-well plate with 50 µL of previously prepared NAR solutions and 200 µL of the ABTS^•+^ solution applied to the wells. The plate was incubated with shaking for 10 min at room temperature. After the incubation, the absorbance values were measured at λ = 734 nm. Vitamin C was used as a standard. The percentage inhibition of the ABTS^•+^ by the samples was calculated according to the following equation:

$$\mathrm{ABTS}\;\mathrm{scavenging}\;\mathrm{activity}\;(\%)=\frac{A_{0}-A_{1}}{A_{0}}\times 100\%$$

where:

*A*_0_ is the absorbance of the control;

*A*_1_ is the absorbance of the sample.

#### 4.2.9. Inhibition of Enzymes Influencing the Development of Neurodegenerative Diseases

The modified spectrometric method developed by Ellman et al. was used to describe the AChE and BChE inhibitory activity [57]. This technique requires artificial substrates (thiocholine esters). Thiocholine is liberated during the enzymatic reactions with 5,5′-dithio-bis-(2-nitrobenzoic) acid (DTNB) and the 3-carboxy-4-nitrothiolate anion (TNB anion) is formed. The enzyme activity is measured spectrophotometrically according to the increase in the color produced by thiocholine. The assay was performed in a 96-well plate. The NAR concentrations for the assay were prepared in the range of 1.0 μg/mL to 6.0 mg/mL in DMSO. The wells contained 60 μL of 0.05 M Tris-HCl buffer, with a pH of 8.0, 20 μL of test solution, and 30 μL of AChE/BChE solution at a concentration of 0.2 U/mL and they were incubated, shaking for 5 min at room temperature. Later, 30 μL of 1.5 mM acetylthiocholine iodide (ATCI)/butyrylthiocholine iodide (BTCI) solution and 125 μL of 0.3 mM DTNB solution (5,5′-dithiobis-(2-nitrobenzoic acid) were added to the well and incubated in the same conditions for 20 min. A blank for the test sample (the reaction mixture was stripped of the enzyme, the volume of Tris-HCl buffer was elevated), the control sample (solvent was pipetted instead of the test sample), and the blank for the control sample (the reaction mixture of the control sample was depleted of the enzyme (the volume of Tris-HCl buffer was elevated)) were also prepared. As a reference, galantamine was used. The absorbance of the test samples was measured at a wavelength of 405 nm. The percentage of inhibition of AChE and BChE by the samples was calculated according to the equation:

$$\mathrm{AChE}/\mathrm{BChE}\;\mathrm{inhibition}\;(\%)=\frac{1-\left(A_{1}-A_{1b}\right)}{\left(A_{0}-A_{0b}\right)}\times 100\%$$

where:

*A*_1_ is the absorbance of the test sample;

*A*_1*b*_ is the absorbance of the blank of test sample;

*A*_0_ is the absorbance of control;

*A*_0*b*_ is the absorbance of the blank of control.

The tyrosinase inhibition assay uses L-DOPA, an amino acid used to replenish dopamine deficiencies, as a substrate for the tyrosinase enzyme. Tyrosinase catalyzes the metabolism of tyrosine to L-DOPA and L-DOPA to dopaquinone. Inhibition of tyrosinase causes a decrease in the degradation of L-DOPA, the precursor of dopamine, which is deficient in Parkinson’s disease patients. The analysis is based on measuring the reduction in color intensity of the solution due to inhibition of enzyme activity [48]. The inhibitor blocks L-DOPA access to the tyrosinase active site, which prevents the reaction from proceeding. The NAR concentrations for assay were prepared in the range of 1.0 μg/mL to 6.0 mg/mL in DMSO. The test sample contained 75 μL 0.1 M phosphate buffer with a pH of 6.8, 25 μL of test solution, and 50 μL of enzyme solution (192 U/mL), and the samples were incubated, shaking at room temperature for 10 min. Later, 50 μL 2.0 mM L-DOPA was added to the well and incubated in the same conditions for another 20 min. A blank for the test sample (the reaction mixture was depleted of the enzyme, the volume of phosphate buffer was increased), the control sample (solvent was introduced instead of the test sample), and the blank for the control sample (the reaction mixture of the control sample was stripped of the enzyme (the volume of phosphate buffer was increased) were also prepared. The absorbance of the test samples was measured at a wavelength of 475 nm. Azelaic acid was used as a standard. The percentage inhibition of the tyrosinase by the samples was calculated using the equation:

$$\mathrm{Tyrosinase}\;\mathrm{inhibition}\;(\%)=\frac{1-\left(A_{1}-A_{1b}\right)}{\left(A_{0}-A_{0b}\right)}\times 100\%$$

where:

*A*_1_ is the absorbance of the test sample;

*A*_1*b*_ is the absorbance of the blank of test sample;

*A*_0_ is the absorbance of control;

*A*_0*b*_ is the absorbance of the blank of control.

#### 4.2.10. Docking

The NAR molecule was drawn manually by using the Avogadro 1.1.1 software and optimized within the UFF force field (5000 steps and steepest descent algorithm) [58,59]. The HP-β-CD molecules were prepared based on the available crystal structures of β-CD and manually substituted by the HP groups, according to the two alternative substitution patterns using Avogadro 1.1.1. Upon this modification, the HP-β-CD structures were optimized using the UFF force field. From the known HP-β-CD molar mass, it was deduced that the most probable number of the HP moieties present in one CD molecule is equal to 6. However, the exact substitution pattern remains unknown. Thus, we have performed calculations for the two alternative compounds: (1) HP-β-CD containing all HP groups substituted to the O_(2)_ hydroxyl oxygen atoms of β-CD (atom numbering in accordance with the IUPAC recommendations); (2) HP-β-CD containing all HP groups substituted to the O_(6)_ hydroxyl oxygen atoms of the hydroxymethyl groups present in β-CD. These two cases represent the two topologically limiting substitution patterns, placing all HP moieties at opposite sides of the β-CD torus. The NAR-HP-β-CD docking was carried out by using the AutoDock Vina software [60]. The procedure of docking was performed within the cuboid region, which covered the whole HP-β-CD molecule. All the default procedures and algorithms implemented in AutoDock Vina were applied during docking. In addition to the flexibility of the ligand molecules, the rotation of the HP groups was allowed as well.

#### 4.2.11. Molecular Dynamics

The MD simulations concerned the NAR-2HP-β-CD complexes. The initial structures of such complexes were based on the most energetically favorable poses identified during the docking study. The MD simulations were carried out within the GROMACS 2016.4 package [61]. The CHARMM force field was applied to describe the interactions within the system, and the CHARMM-GUI online server was used to generate the parameters [62,63]. The considered MD systems consisted of one complex solvated by water molecules (TIP3P model) within a cubic computational box simulated under periodic boundary conditions [64]. The box edges (of initial dimensions corresponding to 5 × 5 × 5 nm^3^) were preoptimized by a 1 ns constant-pressure MD equilibration at 1 bar and 298 K, ensuring an effective solvent density appropriate for these conditions in the subsequent production simulations. After equilibration, all simulations were carried out for 100 ns. The trajectory was saved every 1 ps. The temperature was maintained close to its reference value (298 K) by applying the V-rescale thermostat, whereas for the constant pressure (1 bar, isotropic coordinate scaling) the Parrinello–Rahman barostat was used with a relaxation time of 0.4 ps [65,66]. The equations of motion were integrated with a time step of 2 fs, using the leap-frog scheme [67]. The translational center-of-mass motion was removed every timestep separately for the solute and the solvent. The full rigidity of the water molecules was enforced by the application of the SETTLE procedure [68]. The hydrogen-containing solute bond lengths were constrained by the application of the LINCS procedure with a relative geometric tolerance of 10^−4^ [69]. The electrostatic interactions were modeled by using the particle-mesh Ewald method with the cut-off set to 1.2 nm, while van der Waals interactions (Lennard–Jones potentials) were switched off between 1.0 and 1.2 nm [70].

#### 4.2.12. Statistical Analysis

For statistical analysis, Statistica 13.3 software (StatSoft Poland, Krakow, Poland) was used. Data are presented as mean values ± standard deviations. Experimental data were analyzed using the skewness and kurtosis tests to determine the normality of each distribution, and the Levene’s test assessed the equality of variances. Statistical significance was determined using a one-way analysis of variance (ANOVA) followed by the Bonferroni post hoc test (to compare the experimental results acquired for NAR and NAR in the systems). Differences were considered significant at *p* < 0.05.

## 5. Conclusions

A significant improvement in the solubility of NAR, which is classified as an insoluble compound, is the result of obtaining its dispersions with selected macromolecules. The enlargement in NAR solubility is associated with the improvement of its biological activities, such as antioxidant potential and the possibility of inhibiting the activity of enzymes that are expressed during the development of neurodegenerative diseases.

## Figures and Tables

**Table 1:** Solubility of NAR in ternary solid dispersions. (*) indicates statistically significant differences, *p* < 0.05.

System	Ratio	Method	Solubility (μg/mL)
NAR:HP-β-CD:NaHCO_3_	1.125:3.375:0.5	Co-precipitation, grinding	956.598 ± 13.556 (*)
NAR:HP-β-CD:NaHCO_3_	1:3:1	Co-precipitation, grinding	1156.504 ± 16.163 (*)
NAR:HP-β-CD:Mg stearate	1:3:4	Co-precipitation, grinding	835.502 ± 7.991 (*)
NAR:β-CD:NaHCO_3_	1.125:3.375:0.5	Co-precipitation, grinding	740.775 ± 6.161 (*)
NAR:β-CD:NaHCO_3_	1:3:1	Co-precipitation, grinding	892.559 ± 13.760 (*)
NAR:β-CD:Mg stearate	1:3:4	Co-precipitation, grinding	670.907 ± 4.975 (*)
NAR:HPMC:NaHCO_3_	1.125:3.375:0.5	Co-precipitation, grinding	753.488 ± 9.678 (*)
NAR:HPMC:NaHCO_3_	1:3:1	Co-precipitation, grinding	848.531 ± 11.674 (*)
NAR:HPMC:Mg stearate	1:3:4	Co-precipitation, grinding	771.459 ± 6.007 (*)

**Table 2:** Antioxidant activity of NAR, the binary system (NAR:HP-β-CD) obtained by the co-precipitation method, the physical mixtures (NAR:HP-β-CD; NAR:NaHCO_3_), the ternary system (NAR:HP-β-CD:NaHCO_3_), and the physical mixture (NAR:HP-β-CD:NaHCO_3_). (*) indicates statistically significant differences, *p* < 0.05.

NAR/ph. m./System	Max. Water Conc. (µg/mL)	ABTS ASSAY	CUPRAC ASSAY
		Radical Inhibition (%)	EC
NAR	2.52	0.00	0.00
NAR:HP-β-CD 1:3 ph. m.	506.29	37.43 ± 1.16 (*)	0.44 ± 0.03 (*)
NAR:HP-β-CD 1:3 co-pr.	542.11	38.43 ± 1.23 (*)	0.46 ± 0.04 (*)
NAR:NaHCO_3_ 4:1 ph. m.	278.29	28.73 ± 0.61 (*)	0.29 ± 0.02 (*)
NAR:HP-β-CD:NaHCO_3_ 1:3:1 ph. m.	875.14	45.39 ± 2.01 (*)	0.64 ± 0.04 (*)
NAR:HP-β-CD:NaHCO_3_ 1:3:1 co-pr.; ph. m.	1156.50	49.45 ± 2.41 (*)	0.77 ± 0.05 (*)

## Data Availability

Data are available in a publicly accessible repository.

## Supplementary Materials

The following are available online at https://www.mdpi.com/article/10.3390/ijms23020755/s1.

## Abbreviations

ABTS	2,2′-azino-bis[3-ethylbenzthiazoline-6-sulphonic acid]
AChE	acetylcholinesterase
ANOVA	one-way analysis of variance
API	active pharmaceutical ingredient
ATCI	acetylthiocholine iodide
ATR	attenuated total reflectance
AUC	area under the curve
BBB	blood-brain barrier
BChE	butyrylcholinesterase
BCS	biopharmaceutics classification system
BTCI	butyrylthiocholine iodide
CNS	central nervous system
CUPRAC	cupric ion reducing antioxidant capacity
DAD	diode-array detector
DFT	Density functional theory
DTNB	5,5′-dithio-bis-(2-nitrobenzoic) acid
FRAP	ferric reducing antioxidant power
FT-IR	Fourier-transform infrared spectroscopy
GIT	gastrointestinal tract
HP-β-CD	hydroxypropyl-β-cyclodextrin
HPLC	high-performance liquid chromatography
HPMC	hydroxypropylmethylcellulose
MD	molecular dynamics
NaHCO_3_	sodium bicarbonate
NAR	naringenin
PAMPA	parallel artificial membrane permeability assay
RDF	radial distribution functions
TNB	3-carboxy-4-nitrothiolate
WHO	World Health Organization
XRPD	X-ray powder diffraction
