# Supplementary material for: The Systems of Naringenin with Solubilizers Expand Its Capability to Prevent Neurodegenerative Diseases
Source: Int J Mol Sci. 2022 Jan 11;23(2):755. doi: 10.3390/ijms23020755 (PMC8775867; doi:10.3390/ijms23020755)
Supplement: Supplementary file 1 [file ijms-23-00755-s001.zip › ijms-1518147-supplementary.pdf]

International Journal of
Molecular Sciences

MDPI

Supplementary Materials

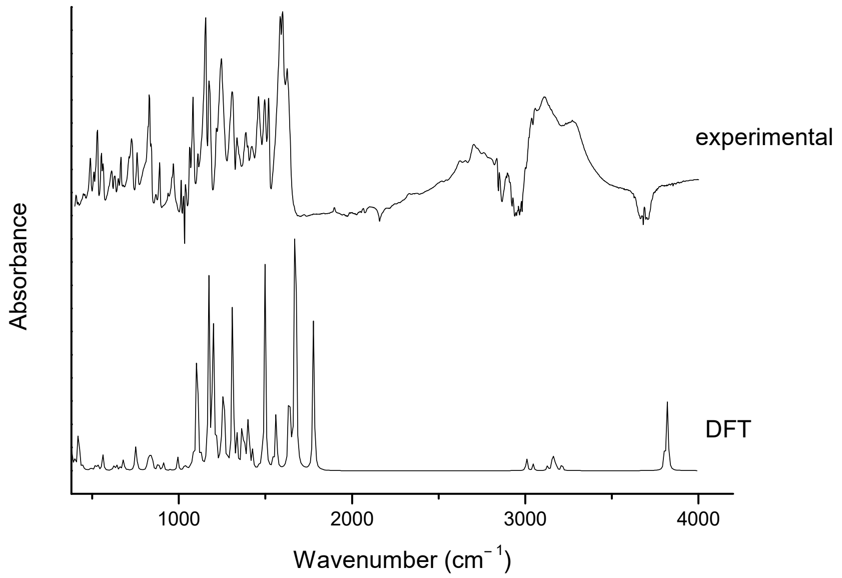

Figure S1. Calculation (DFT) and experimental IR absorption spectra of NAR at room temperature.

Table S1. Selected characteristic vibronic features of curcumin theory with application of 6-31G(d,p) basis and experiment bands of NAR. s-stretching, b-bending, w-wagging, r-rocking, t-twisting, def. - deformation.

Calculation (cm^{-1})	Experiment (cm^{-1})	Band Assignment
562	563	Def. all molecule
751	759	C-H w
832	831	C-H w
1030	1038	C-H w
1070	1063	CH_2 t + C-O s
1082	1081	C-C s
1105	1109	C-O s
1172	1154	C-H r + C-O-H b
1201	1175	C-H r + C-O-H b
1259	1245	C-H r + C-O s + CH_2 t + C-O-H b
1426	1421	C-O s
1498	1496	H-C=C b
1563	1600	C=C s
1779	1626	C=O s
3007	2900	C-H s
3046	3035	C-H s
3166	3115	C-H s
3805	3688	O-H s
